# Reduction of *Stromatinia cepivora* inocula and control of white rot disease in onion and garlic crops by repeated soil applications with sclerotial germination stimulants

**DOI:** 10.1016/j.heliyon.2019.e01168

**Published:** 2019-01-30

**Authors:** Ibrahim E. Elshahawy, Ahmed A. Morsy, Farid Abd-El-Kareem, Nehal M. Saied

**Affiliations:** Plant Pathology Department, Agricultural and Biological Division, National Research Centre, Giza, Egypt

**Keywords:** Biotechnology, Microbiology, Plant biology

## Abstract

The effect of soil-applied *Allium* crop products on the *Stromatinia cepivora* viability and the incidence of white rot in subsequent onion and garlic crops were evaluated in this work. The tested products were onion powder, garlic powder, onion oil, garlic oil and *Allium* waste (onion and garlic) that are described as sclerotial germination stimulants. Under *in vitro* conditions, data revealed that more than 80% of the sclerotia died in the soil treated with sclerotial germination stimulants. Under greenhouse conditions, soil-artificially infested with sclerotia of *S. cepivora* and treated with sclerotial germination stimulants for 6-months before cultivation, significantly reduced the incidence of white rot on onion and garlic. Onion oil, garlic oil and *Allium* waste were the most effective treatments, decreasing disease incidence by 78.6% in onion and 80.0% in garlic. Under field conditions, sclerotial germination stimulants were incorporated into the soil in commercial fields naturally infested with *S. cepivora*. Two fields were chosen based on differential sclerotial density. Within 6 months after treatment, more than 70% of the sclerotia died in the plots treated with sclerotial germination stimulants. In subsequent onion and garlic crops planted approximately one year after soil treatment, sclerotial germination stimulants were more effective than the control in reducing white rot symptoms coupled with low inoculum density (45.9 sclerotia/kg of soil). Reduction of white rot disease was accompanied by increased of growth and bulbs yield of onion and garlic plants. Despite the efficacy of sclerotial germination stimulants to reduce populations of viable sclerotia in soil with a high inoculum density (594.7 sclerotia/kg of soil), the pathogen caused substantial white rot and yield losses in subsequent onion and garlic crops planted approximately one year after soil treatment.

## 1 Introduction

Onion (*Allium cepa* L.) and garlic (*Allium sativum* L.) are among the most important vegetable crops in Egypt for local consumption and exportation. Approximately 80,000 and 10,500 hectares were cultivated in 2015/2016 yielding 2,888,791 and 246991 metric tons, respectively (Ministry of Agriculture of Egypt, 2017). White rot disease caused by the soil borne fungus *Stromatinia cepivora* (Berk.) Whetzel is a serious threat to onions and garlic worldwide and is a major limiting factor in the production of both crops in Egypt (Hunger et al., 2002; Metcalf et al., 2004; Elshahawy et al., 2017a,b,c; Elshahawy et al., 2018a,b). The first reference of white rot in Egypt was in 1922. Since then, the incidence of disease has risen progressively and nowadays the disease rate can exceed 80.0% in some commercial onions and garlic fields resulting in significant yield losses (Elshahawy et al., 2018a,b). Loss estimates are difficult to ascertain because once the disease is identified in a field, growers are forced to grow other non susceptible (non-*Allium*) crops. Further onion or garlic production frequently does not occur in infested fields. *S. cepivora* propagates by the production of small (0.3–0.6 mm in diameter), round, poppy-seed-sized sclerotia on the roots of decayed host plants. Sclerotia spread via mass movement of soil or water and especially trough the infested plant parts. The sclerotia are stimulated to germinate only by *Allium*-specific root exudates, indicating that the host range is limited to *Allium* species (Amin et al. 2014). The severity of white rot is usually related directly to the number of sclerotia in the soil at planting (Crowe et al., 1980). However, few sclerotia can result in great crop losses; for example, economic losses due to white rot can occur at inoculum densities as low as 0.1 sclerotium/L of soil and populations above 10 sclerotia/liter of soil may cause near total crop loss. Populations near 1 sclerotium/L of soil may cause crop losses between 30 and 60% (Crowe et al. 1980). When a sclerotium germinates and infects an *Allium* root, mycelia grow upward towards the bulb, eventually destroying it (Crowe et al., 1980). The fungus also spreads from plant to plant, increasing disease incidence within the same growing season.

Management of *S. cepivora* using the crop rotation method is ineffective because it forms abundant sclerotia that can remain dormant for several years (Crowe et al., 1993). Breeding for resistance has not been particularly successful, possibly because the development of resistant cultivars is difficult and time consuming. Furthermore, developing a new variety having with appropriate bulb quality and disease resistance is difficult (Earnshaw et al., 2000). Other methods used to reduce *Allium* white rot severity such as, fungicides (Zewide et al., 2007), soil flooding (Banks and Edgington, 1989), addition of composts (Entwistle, 1990), solarization and mulching (Satour et al., 1989), incorporation of cruciferous residues (Smolinska, 2000), application of antagonists (Elshahawy et al., 2017a), and sclerotial mycoparasites (Elshahawy et al., 2017b), have been found to be moderately effective to varying degrees (Prados-Ligero et al., 2002). An effective control method has been obtained with soil partial-sterilizers such as methyl bromide, dazomet and vapam which destroy the sclerotia (Adams and Johnston, 1983), but such treatments are expensive. In addition, the materials themselves are scheduled to be phased out for use according to the Environmental Protection Agency, which initiated action under the clean air act for a phase-out of chemicals threatening the ozone layer (Adams and Johnston, 1983).

Alternative approaches to managing white rot disease have focused on reducing the populations of sclerotia of *S. cepivora* by stimulating sclerotial germination in the absence of onion and garlic crops (Coley-Smith and Parfitt, 1986; Davis et al., 2007). Sclerotia of *S. cepivora* can survive for at least 40 years in the absence of specific sulfides exuded from roots of *Allium* crops and can act as a source of infection (Coley-Smith, 1960). The root exudates of *Allium* crops contains alkyl and alkenyl-L-cysteine sulphoxides, which is transformed by soil microorganisms into volatile thiols and sulfides, which in turn activates the dormant sclerotia (Coley-Smith, 1960). Through this specificity between the sclerotia of *S. cepivora* and the root exudates of *Allium* crops, volatile thiols and sulfides can be used to manage white rot disease. If the soil is treated with these thiols in the absence of the *Allium* crop, the sclerotia grows in the absence of a host. Mycelium germinated from the sclerotia cannot compete with the microorganisms found in the soil to recover on the organic material. It lives after germination for a short period depends on the appropriate temperature and the amount of self-food and therefore if not found a host will die. The compound diallyl disulphide (DADS) is present naturally in *Allium* spp., and its products such as onion oil, garlic oil and others, and is considered a natural sclerotial stimulant if applied artificially in the soil (Coley-Smith, 1990; Davis et al., 2007). When applying this compound to the soil in the absence of *Allium* crop, about 90–99% of sclerotia will germinate (Tyson et al., 2000; Hovius and McDonald, 2002; Davis et al., 2007). Such stimulation is also possible using natural preparations from *Allium* spp. (Coley-Smith and King, 1969; Davis et al., 2007). In Australia, Merriman et al. (1980) used natural onion oil to stimulate sclerotial germination in the field. They found reductions of approximately 60% of sclerotial populations in the field using artificial onion oil as a source of stimulant, but economic control of the disease was only obtained when the final sclerotial population was reduced to ∼50 sclerotia/kg. In the Netherlands, Merriman et al. (1981) reported that field plots treated with onion oil and diallyl disulphide reduced recovery of introduced sclerotia by 70%. In California, Davis et al. (2007) used garlic powder, synthetic garlic oil and DADS in commercial fields naturally infested with *S. cepivora*. They found that the degree of sclerotial mortality in plots treated with garlic powder at 112 kg/ha or higher was almost equal to that achieved by DADS at 500 ml/ha or methyl bromide at 448 kg/ha. Little information has been cited in the literature on the possibility of using stimulants of sclerotial germination to control white rot disease in onions and garlic in Egypt. Therefore, the objective of this study was to evaluate the impact of each germination stimulant used in the absence of *Allium* crops on the density and viability of sclerotia of *S. cepivora*, as well as the incidence of white rot and onion and garlic yield under field conditions.

## 2 Methods

### 2.1 Sclerotial germination stimulants

Germination stimulants materials, *i.e.,* onion powder, garlic powder, onion oil, garlic oil and *Allium* wastes, were obtained from El-Nenaiea Company for Industry and Trading for Production of Dehydrated Vegetable and Fruits, Kafr Abu Mahmoud, Ashmoun, Menofia, Egypt. These materials were standard grade onion and garlic powders (the purest commercial product is made by drying and granulating fresh onion and garlic), naturally extracted onion oil, naturally extracted garlic oil and *Allium* waste (ground peels of onion and garlic).

### 2.2 Fungal isolates and production of sclerotia

Two isolates of *S. cepivora* (*S.cepivora* Sc2-isolated from infected onion plants and *S. cepivora* Sc8-isolated from infected garlic plants) were obtained from the author's collection. These isolates were classified as highly virulent against onions and garlic based on pathogenicity tests conducted in previous studies (Elshahawy et al., 2017a, 2018b). Sclerotia were produced by infection of onion and garlic bulbs with mycelium of *S. cepivora* Sc2 and *S. cepivora* Sc8, grown on potato dextrose agar PDA (Coley Smith, 1985). The bulbs were incubated in moist sand at 15 °C. The newly formed sclerotia were harvested after approximately 5 weeks, mixed with sand (10 sclerotia per gram of sand) and stored in nylon mesh bags (= 0.1 mm mesh size, 10 × 10 cm^2^) in soil for 8 weeks at 20 °C followed by 8 weeks at 5 °C (Gerbrandy, 1992). Each bag was filled with approximately 10 grams and 5 grams of the mix of sclerotia-sand of each isolate.

### 2.3 Laboratory experiment

Sandy-loam soil was used in the experiments. Soil was amended with 0.1% of each onion powder, garlic powder, onion oil, garlic oil and *Allium* waste and put individually into a glass jar (volume1 L). Three bags made of nylon netting containing 100 sclerotia each, were placed in the middle of the jar. Three jars with three bags in each of them were prepared for each treatment. Soil without amendments containing bags of sclerotia was used as a control. Soil in the jars was wetted to 25% at the field capacity. After 120 days of incubation at 18 ± 2 °C, the sclerotia were removed from the soil and counted under a dissecting microscope. The sclerotia recovered from the bags were surface sterilized in 0.25% NaOCl for 1 min, washed in five changes of sterile distilled water and blotted dry on sterile Whatman no. 1 filter paper. The sclerotia were then placed individually using sterile forceps onto isolated PDA droplets in Petri dishes and were incubated in the dark at 18±2 °C for 10 days. The percentage viability of sclerotia, relative to the number buried was determined. Three replicates were prepared per treatment, and the experiment was repeated. Data were analysed statistically by the analysis of variance test (ANOVA) and the means were compared by Duncan's multiple range test at *P* < 0.05. Percent data were statistically analysed after arcsine square-root transformation; however, untransformed data are presented.

### 2.4 Greenhouse experiment

#### 2.4.1 Effect of germination stimulants on sclerotial viability

Plastic containers (40 × 40 × 20 cm) were filled with coarsely sieved, air-dried, unsterile, free of *S. cepivora* sclerotia sandy-loam soil. This soil was infested with sclerotial inocula (100 sclerotia/kg soil) of *S. cepivora* isolates according to the method of Metcalf et al. (2004). Soil was then amended with 0.1% of each onion powder, garlic powder, onion oil, garlic oil and *Allium* waste, then wetted to 25% and maintained throughout the course of the trial. Soil without amendments was used as a control. The effect of germination stimulants on sclerotia was determined. In brief, three nylon mesh bags containing 100 sclerotia were buried in each plastic container at a depth of 10 cm. Six plastic containers were maintained for each stimulant as well as the control. The experiment was carried out on 1 December 2016, under greenhouse conditions at 20 ± 2 °C maximum and 10 ± 2 °C minimum. For each treatment, bags of sclerotia were removed 6 months after application and the percentages of viable sclerotia relative to the number buried were determined as mentioned before.

#### 2.4.2 Effect of germination stimulants on white rot incidence

After removal of bags containing sclerotia, soil found in plastic container were transferred to sterilized plastic pots (30 cm-diameters), wetted and maintained under greenhouse conditions for further investigation. Garlic cloves were sown on 10 October and onion seedlings were sown on 1 December 2017. Five garlic cloves (cv. Sides 40) and five seedlings (60- days old) of onion (cv. Giza red) were sown in pots. For each crop, three plastic pots were used as replicates for each treatment as well as the control of each crop. The number of plants with white rot symptoms and their percentage was evaluated 100 days later. The fresh weight of onion and garlic plants from each pot of the different treatments was also recorded after harvest as g/pot. Data were statistically by the analysis of variance test (ANOVA) and the means were compared by Duncan's multiple range test at *P* < 0.05. Percent data on disease incidence were statistically analysed after arcsine square-root transformation; however, untransformed data are presented.

### 2.5 Field experiment

#### 2.5.1 Selection of trial locations

Field trials were located in El-Deer village, El-Qalubia governorate, in which white rot disease is currently of interest. In this region, several fields with a well-established history of white rot disease were sampled preliminarily for inoculum density determinations according to the procedure of Utkhede and Rahe (1979). After that, two field sites were chosen. One of them was characterized by its low sclerotial density with an average of 45.9 sclerotia/kg of soil. The second was characterized by high sclerotial density with an average of 594.7 sclerotia/kg of soil. To avoid constitutive dormancy of sclerotia, non-*Allium* crops were grown in each field for two years before each experiment was initiated. Our experiment was performed in the period from December 2016 to May 2018 and involved two phases. The first phase (December 1, 2016–May 1, 2017) aimed to estimate the efficiency of germination stimulants used in the absence of *Allium* crops on the density and viability of sclerotia of *S. cepivora*. The second phase (September 15, 2017–May 15, 2018) aims to estimate the efficiency of the above treatments on the incidence of white rot in the subsequent growing onion and garlic crops as well as growth parameters and yields of onion and garlic under field conditions.

#### 2.5.2 Application of sclerotial germination stimulants

In each field trial, the experimental area was divided into six blocks each measuring 3.0 × 57.0 m with 6.0 m margins in between. Each block was divided into six plots, each measuring 3.0 × 3.5 m. In all cases, plots were separated by a 6-m border to prevent cross contamination of the volatile compounds. On these plots, the following treatments were carried out at random: (1) control (no stimulant amendment), (2) onion powder amendment, (3) garlic powder amendment, (4) *Allium* waste amendment, (5) onion oil and (6) garlic oil. The experimental design consisted of randomized block designs with six replications per treatment. The solid stimulants were applied to the soil in field plots at a rate of 1000 kg/ha while liquid stimulants were applied at a rate of 10 L/ha. All stimulants were applied three times: 1/12/2016, 1/2/2017 and 1/4/2017. At the time of each application, the amount of stimulants per plot area was premixed using an equal amount of non-ionic wetting agent (sand) to facilitate mixing. Wetting agent without stimulants as well as spaded and/or rotilled plots served as controls. After each application, soil in plots was spaded and/or rotilled to a depth of 15 cm to encourage product infiltration followed by flood irrigation. During these operations, soil among plots remained unaltered. To reduce the loss of volatilization agents, the soil is irrigated 15–30 minutes after application. Soil moisture is then maintained unless there is rain (Crowe and Hall, 1980). In general, the soil is irrigated when moisture is reduced to below the field capacity, especially when soil temperatures are conducive to stimulate the sclerotia. The soil was not moved from plot to another, in addition to preventing the passage of washing water during irrigation from one plot to another by constructing dams between the plots. Weed control was done using commercially available herbicides while avoiding soil disturbance during spraying.

#### 2.5.3 Effect of germination stimulants on sclerotial viability

The inoculum density of viable sclerotia of *S. cepivora* was determined from each plot in each field trial immediately prior to each treatment with germination stimulants and after two months of the last application. Therefore, soil samples were collected four times: 30/11/2016, 31/1/2017, 29/3/2017 and 30/5/2017. In the second phase, soil samples were collected twice: before sowing garlic (14-September 2017) and onion (31-November 2017). To determine the number of viable sclerotia, a wet-saving floatation technique that developed by Utkhede and Rahe (1979) was used. Samples were collected by taking twenty soil cores of approximately 300 g from the upper 15 cm of soil along each diagonal of diamond pattern and were combined into three composite samples for sclerotia assay. The soil samples were dried in open plastic bags at room temperature (25 °C) until weight loss stabilized. Soil samples (100 g) were passed through two stacked sieves (0.595-mm openings over 0.210-mm openings) for at least 5 min under a spray of water. The residues on the 0.210 mm sieves were transferred to columns containing 2.5 M sucrose solution. After 2 hours the soil fractions suspended in the upper portions of the columns were collected, washed with water on 0.210-mm sieves and examined with a dissecting microscope. The obtained sclerotia were removed with forceps and were then surface sterilized in 0.25% sodium hypochlorite for 3 min, washed in distilled water and dried between folds of sterilized filter papers. Surface-sterilized sclerotia were subsequently plated on potato dextrose agar Petri dishes. Each sclerotium was cracked (by pinching with forceps) during placement to induce rapid and simultaneous germination (Crowe et al., 1980). Petri dishes maintained at 20±2 °C for 2 weeks were examined at ×10 with a stereo microscope. Inoculum densities were expressed as the number of viable sclerotia per kilogram of air-dried soil. Data were analysed using analysis of variance (ANOVA) with the following factors: germination stimulants and time of application.

#### 2.5.4 Effect of germination stimulants on white rot incidence

Based on garlic and onion production regimes, the plots were prepared for garlic planting on 15-September 2017 and onion planting on 1-December 2017. The soil of each plot was hand spaded and tilled. For each crop, three replicated plots were used for each germination stimulant as well as untreated controls. The plot area was 3.0 × 3.5 m and each plot included 6 rows (each measuring 3.0 m in length and 50 cm in width). Garlic cloves cv. Sides 40 that had been uniformly sized were hand planted 5 cm deep in rows spaced 10 cm × 10 cm within each row. Additionally, 60-day-old onion transplants (cv. Giza red) were transplanted in each row at a spacing of 10 cm × 10 cm. Garlic and onion were grown to maturity under irrigation, fertilization and pest management practices standard with commercial production in the area. White rot disease evaluations were conducted periodically during the 2017/2018 season based on top symptoms of white rot, and were confirmed by gently removing some soil from around the base of some plants. At harvest, bulbs with symptoms of white rot were assessed by pulling and observing all garlic and onion bulbs in each plot. The percentage of infected plants as well as white rot reduction (%) was calculated according to Elshahawy et al. (2017a) as follows:

$$\text{White}\;\text{rot}\;\text{infection}\left(\%\right)=\frac{\text{Number}\;\text{of}\;\text{infected}\;\text{plants}\;\text{with}\;\text{white}\;\text{rot}}{\text{Total}\;\text{number}\;\text{of}\;\text{plants}}\times 100$$

$$\text{Whit}\;\text{rot}\;\text{infection}\left(\%\right)=\frac{\text{White}\;\text{rot}\left(\text{\%}\right)\text{in}\;\text{control}-\text{White}\;\text{rot}\left(\text{\%}\right)\text{in}\;\text{treatment}}{\text{White}\;\text{rot}\left(\text{\%}\right)\text{in}\;\text{control}}\times 100$$

#### 2.5.5 Effect of germination stimulants on plant growth and bulb yield

For each crop, at 100 days after planting, some vegetative growth parameters, including average plant height (cm), average number of leaves/plant and average plant biomass (g) were estimated. As bulbs matured, bulbs of garlic and onion were harvested and weighed (kg/plot) for yield assessment. Data collected from field experiments were analysed separately for each crop. Data was analysed using an analysis of variance (ANOVA). The means were compared by Duncan's multiple range test at *P* < 0.05. Percent data on disease incidence were statistically analysed after arcsine square-root transformation; however, untransformed data are presented.

#### 2.5.6 Benefit-cost balance of treatments

Costs and benefits of the different sclerotial germination stimulants applied for controlling white rot disease of onion and garlic plants under low sclerotial density (45.9 sclerotia/kg of soil) and high sclerotial density (594.7 sclerotia/kg of soil) were calculated according to Bulluck (1988).

## 3 Results

### 3.1 Survival of sclerotia of *S. cepivora*

#### 3.1.1 Laboratory trial

Sclerotia of *S. cepivora* exposed to germination stimulants resulted in reduced survival of viable sclerotia after 120 days of exposure (Table 1). At the end of the experiment, 85.0% of sclerotia had germinated in the glass jars without germination stimulants, compared with between 4.0 and 18.3% for the germination stimulants treatments. Onion oil, garlic oil and *Allium* waste were the most effective treatments in reducing sclerotia survival. After 120 days of exposure to these treatments, the survival of viable sclerotia was reduced to 91.3, 92.7 and 96.0 %, respectively, compared with 15.0% in untreated soil. Onion powder and garlic powder showed moderate effects reducing the survival of viable sclerotia by 83.0 and 81.7%, respectively.

**Table 1:** Percentage of *S. cepivora* sclerotial recovery and their viability from all treatments of sclerotial germination stimulants after 120 days incubation under laboratory conditions.

Stimulant	Sclerotial recovery and sclerotial viability (%)(a)	
	% recovery	% viability
Non-amended control	92.0 ± 1.2 a(b)	85.0 ± 1.7 a
Onion powder	23.0 ± 1.5 c	17.0 ± 0.6 b
Garlic powder	25.0 ± 1.0 b	18.3 ± 0.3 b
Onion oil	16.3 ± 0.9 d	8.7 ± 1.8 c
Garlic oil	10.7 ± 0.7 e	7.3 ± 0.3 cd
*Allium* waste	18.0 ± 0.6 d	4.0 ± 0.6 d

(a) The percentages of rind intact, body firm, no germinating sclerotia and germination percentage were calculated and compared to non-treated control at zero time. Germination of sclerotia at zero time was 100%.

(b) Means ± standard errors within a column followed by the same letter are not significantly different by Duncan multiple range test at *P* < 0.05.

#### 3.1.2 Greenhouse trial

After 6 months of application, soil treated with germination stimulants at the rate of 0.1% lowered the recovery of viable, intact sclerotia compared with the untreated control (Table 2). Performance was better in soil treated with *Allium* waste followed by onion oil and garlic oil than in that treated with other treatments. After 6 months of exposure to these treatments, the survival of viable sclerotia was reduced to 99.3, 93.0 and 92.3 %, respectively, compared with 11.7% in untreated soil. Onion powder and garlic powder showed moderate effects, reducing the survival of viable sclerotia by 88.3 and 90.0%, respectively.

**Table 2:** Effect of different germination stimulants on white rot inoculum density, disease incidence and yields (g/pot) in soil artificially infested with *S. cepivora* sclerotia after 6 months incubation under greenhouse conditions.

Treatment	Viable sclerotia at planting(a)	White rot incidence (%)		Yield (g/pot)	
		Onion	Garlic	Onion	Garlic
Control	88.3 ± 0.9 a(b)	93.3 ± 6.7 a	100.0 ± 0.0 a	49.7 ± 1.2 d	57.0 ± 0.6 d
Onion powder	11.7 ± 0.9 b	40.0 ± 0.0 b	46.0 ± 0.7 b	150.9 ± 1.1 c	156.3 ± 1.4 c
Garlic powder	10.0 ± 0.7 b	40.0 ± 0.0 b	40.0 ± 0.0 b	148.5 ± 0.3 c	154.7 ± 1.8 c
Onion oil	7.0 ± 0.6 c	20.0 ± 0.0 c	20.0 ± 0.0 c	170.7 ± 0.7 b	177.3 ± 9.3 b
Garlic oil	7.7 ± 0.9 c	20.0 ± 0.0 c	20.0 ± 0.0 c	170.0 ± 3.1 b	187.0 ± 1.2 b
*Allium* waste	0.7 ± 0.7 d	20.0 ± 0.0 c	20.0 ± 0.0 c	193.7 ± 2.0 a	246.3 ± 0.7 a

(a) The number of germinating sclerotia was counted at 6 months after application and compared with the number of germinating sclerotia before application. Germination of sclerotia at zero time was 100%.

(b) Means ± standard errors within a column followed by the same letter are not significantly different by Duncan multiple range test at *P* < 0.05.

#### 3.1.3 Field trials

**Fig. 1:**
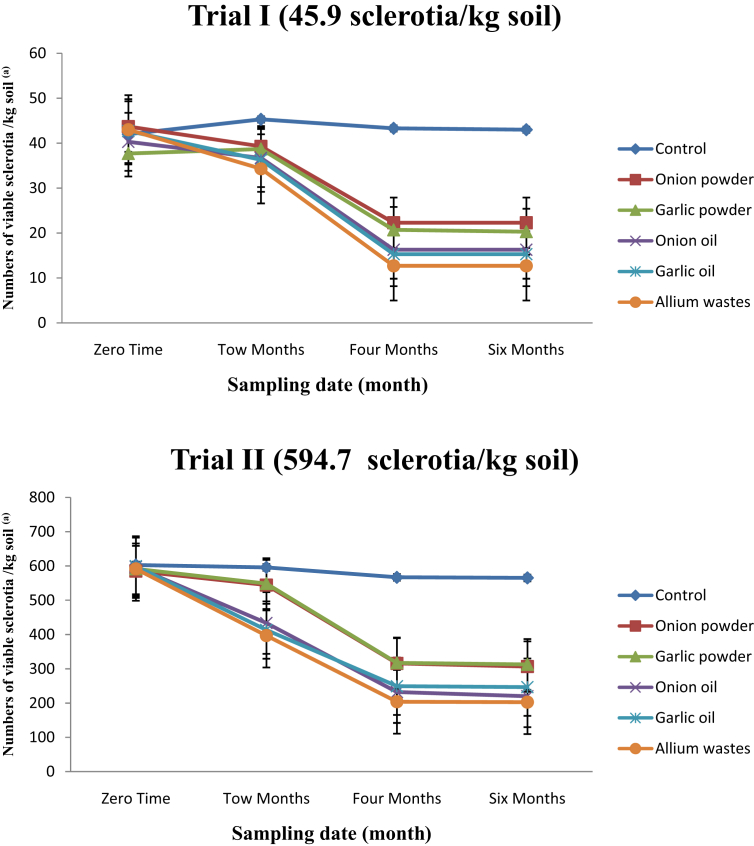
Numbers of viable sclerotia of *S. cepivora* in 1 kg of soil sampled immediately before treatments were applied (the first sampling date in each trial) and periodically thereafter. Sclerotial germination stimulants were added to soil in 1-December-2016. ^(a)^ Inoculum densities were expressed as the number of viable sclerotia per kilogram of air-dried soil. Each bar represents the standard error of the mean.

The application of the tested materials significantly increased sclerotial germination compared with the control treatment (Table 3). The results of the population dynamics of viable, intact sclerotia of *S. cepivora* when the tested materials were applied in trials with low inoculum density are shown in Table 3 and Fig. 1. Initial populations of viable sclerotia in soil at the time of the first application were 45.9 sclerotia/kg of soil. Thereafter, during the period of the experiment, the population of viable, intact sclerotia declined progressively and at 6 months after initial application, a significant reduction of more than 50.4–71.7% was recorded. Among the substrates tested, the *Allium* waste treatment proved to be the best at all sampling dates, followed by garlic oil, onion oil, garlic powder and onion powder. The results of the population dynamics of viable, intact sclerotia of *S. cepivora* when the tested materials were applied in trials with high inoculum density (594.7 sclerotia/kg of soil) show that there was also a progressive decline of *S. cepivora* from 2 months to 6 months after the initial application. Still, the population recovered at each sampling date was much greater than that recorded during the same period under trials with low inoculum density. At 6 months after the initial application, a significant reduction of more than 47.8–66.3% was recorded. Among the substrates tested, *Allium* wastes proved to be the best at all sampling dates. This treatment was followed by garlic oil, onion oil, garlic powder and onion powder. The results of the effect of different substrates on the population dynamics of viable, intact sclerotia of *S. cepivora* at the planting time are shown in Tables 4 and 5. Data indicate that an additive reduction in the population of viable, intact sclerotia was recorded at planting date of each crop. The *Allium* waste treatment supported the highest reduction of viable, intact sclerotia by 94.3 and 79.3% on 14-September 2017 and by 96.8 and 81.2% on 31-November 2017, in low and high inoculum densities trials, respectively, followed by the onion oil and garlic oil treatments. On the other hand, onion powder and garlic powder were found to moderately efficient, resulting in the lowest reduction of viable, intact sclerotia.

**Table 3:** Numbers of viable sclerotia of *S. cepivora* in 1 kg of soil sampled immediately before treatments were applied (the first sampling date in each trial) and periodically thereafter.

Treatment	Viable sclerotia/kg soil			
	Sampling date (month)			
	0.0	2.0	4.0	6.0
**Trial I (45.9 sclerotia/kg soil)**				
Control	48.0 ± 0.6 a	45.3 ± 1.3 a	43.3 ± 0.9 a	43.0 ± 0.6 a
Onion powder	43.7 ± 1.4 a	38.7 ± 0.7 bc	20.7 ± 0.7 b	20.3 ± 0.3 c
Garlic powder	43.3 ± 2.4 a	39.3 ± 0.7 b	22.3 ± 0.3 b	22.3 ± 0.3 b
Onion oil	46.7 ± 0.9 a	36.7 ± 0.9 bcd	16.3 ± 0.7 c	16.3 ± 0.7 d
Garlic oil	46.0 ± 1.2 a	36.3 ± 0.3 cd	15.3 ± 0.3 c	15.3 ± 0.3 d
*Allium* waste	47.7 ± 0.3 a	34.3 ± 0.3 d	12.7 ± 0.3 d	12.7 ± 0.3 e
**Trial II (594.7 sclerotia/kg soil)**				
Control	603.0 ± 4.5 a	596.0 ± 3.3 a	567.3 ± 0.9 a	565.3 ± 0.9 a
Onion powder	585.3 ± 7.7 a	544.3 ± 5.4 b	316.0 ± 2.3 b	306.7 ± 3.3 b
Garlic powder	591.7 ± 3.2 a	548.7 ± 0.7 b	317.3 ± 0.3 b	313.0 ± 1.0 b
Onion oil	597.0 ± 15.4 a	433.7 ± 10.7 c	232.3 ± 13.9 c	220.0 ± 15.3 cd
Garlic oil	599.3 ± 0.7 a	413.3 ± 6.8 d	249.3 ± 13.5 c	246.7 ± 12.0 c
*Allium* waste	591.7 ± 1.3 a	397.0 ± 1.7 d	204.0 ± 4.0 d	202.7 ± 3.7 d

Source of variation	*df*		Mean square	
	Trial I	Trial II	Trial I	Trial II
Blocks	2	2	1.0138889^ns^	105.79529^ns^
Time	3	3	2674.1481^∗∗∗^	344048.28^∗∗∗^
Stimulants	5	5	523.12222^∗∗∗^	80812.569^∗∗∗^
Time × stimulants	15	15	89.581481^∗∗∗^	12410.717^∗∗∗^
Error	46	45	2.2167874	172.45354

Ns, not significant; ***P < 0.05.

**Table 4:** Effects on white rot incidence (as percent of infected plants) in garlic due to application of sclerotial germination stimulants in the absent of *Allium* crops during the previous season under field conditions.

Treatment	Viable sclerotia/kg soil(a)		Garlic white rot	
	Before application	At planting	Infected plants (%)	Reduction (%)
**Trial I (45.9 sclerotia/kg soil)**				
Control	48.0 ± 0.6 a(b)	34.3 ± 2.2 a	47.0 ± 1.5 a	-
Onion powder	43.7 ± 1.5 a	13.3 ± 0.7 b	29.3 ± 0.7 c	37.7
Garlic powder	43.3 ± 2.4 a	12.3 ± 0.3 b	32.0 ± 1.0 b	31.9
Onion oil	46.7 ± 0.9 a	4.3 ± 0.3 c	17.3 ± 0.7 d	63.2
Garlic oil	46.0 ± 1.2 a	4.7 ± 0.7 c	18.3 ± 0.3 d	61.1
*Allium* waste	47.7 ± 0.3 a	2.3 ± 0.3 c	13.0 ± 1.0 e	72.3
**Trial II (594.7 sclerotia/kg soil)**				
Control	603.0 ± 4.5 a	462.0 ± 22.2 a	86.3 ± 0.9 a	-
Onion powder	585.3 ± 7.3 a	259.7 ± 21.5 b	82.3 ± 0.9 bc	4.6
Garlic powder	591.7 ± 3.2 a	241.0 ± 12.5 b	84.0 ± 1.2 ab	2.7
Onion oil	597.0 ± 15.4 a	168.3 ± 8.1 c	81.0 ± 0.6 c	6.1
Garlic oil	599.3 ± 0.7 a	137.0 ± 9.8 c	82.7 ± 0.3 bc	4.2
*Allium* waste	591.7 ± 1.3 a	124.7 ± 0.7 c	80.6 ± 0.7 c	6.6

(a) Sclerotial germination stimulants were added to soil in 1-12-2016. The garlic was sown in September 15, 2017.

(b) Means ± standard errors within a column followed by the same letter are not significantly different by Duncan multiple range test at *P* < 0.05.

**Table 5:** Effects on white rot incidence (as percent of dead plants) in onion due to application of sclerotial germination stimulants in the absent of *Allium* crops during the previous season under field conditions.

Treatment	Viable sclerotia/kg soil^a^		Onion white rot	
	Before application	At planting	Infected plants (%)	Reduction (%)
**Trial I (45.9 sclerotia/kg soil)**				
Control	48.0 ± 0.6 a(b)	33.0 ± 1.5 a	42.3 ± 1.2 a	-
Onion powder	43.7 ± 1.5 a	10.0 ± 1.0 b	10.7 ± 0.7 b	74.0
Garlic powder	43.3 ± 2.4 a	9.0 ± 0.6 b	11.0 ± 0.6 b	73.9
Onion oil	46.7 ± 0.9 a	2.0 ± 0.0 c	8.7 ± 0.3 bc	79.4
Garlic oil	46.0 ± 1.2 a	1.7 ± 0.3 c	8.0 ± 0.6 c	81.1
*Allium* waste	47.7 ± 0.3 a	1.3 ± 0.3 c	4.7 ± 0.3 d	88.9
**Trial II (594.7 sclerotia/kg soil)**				
Control	575.7 ± 5.5 a	451.7 ± 27.7 a	82.3 ± 1.2 a	-
Onion powder	544.0 ± 22.3 ab	249.0 ± 24.8 b	77.3 ± 0.3 b	6.1
Garlic powder	527.7 ± 8.9 b	231.7 ± 15.8 b	75.3 ± 0.3 bc	8.5
Onion oil	559.0 ± 17.4 ab	155.7 ± 5.7 c	73.7 ± 1.2 bc	10.4
Garlic oil	557.0 ± 5.3 ab	130.0 ± 7.0 c	72.3 ± 1.2 c	12.2
*Allium* waste	576.0 ± 6.4 a	113.3 ± 1.3 c	71.7 ± 0.9 c	12.9

(a) Sclerotial germination stimulants were added to soil in 1-12-2016. The onion was sown in December 1, 2017.

(b) Means ± standard errors within a column followed by the same letter are not significantly different by Duncan multiple range test at *P* < 0.05.

### 3.2 White rot disease incidence

#### 3.2.1 Greenhouse trial

After 6 months of application, soil treated with germination stimulants at the rate of 0.1% lowered the incidence of white rot on onion and garlic compared with the untreated control (Table 2). Onion oil, garlic oil and *Allium* waste treatments were the most effective, with the lowest percentage of disease incidence (20.0% in onion and garlic), in comparison with 93.3% and 100% for the control in onion and garlic, respectively. Onion powder and garlic powder showed moderate effects.

#### 3.2.2 Field trials

The results of the two trials followed the same trends, but the amount of white rot was related to the inoculum density. The mean disease incidence of *S. cepivora* infection among plants in soil containing 45.9 sclerotia/kg of soil was significantly lower than among those in soil containing 594.7 sclerotia/kg of soil. In general, the germination stimulants were more effective in reducing white rot disease in the trial with low inoculum density than in that with high inoculum density. In the low inoculum density trial, the *Allium* waste treatment was the most effective, with the lowest disease incidence (4.7% in onion and 13.0% in garlic), compared with 42.3% in onion and 47.0% in garlic for the controls (Tables 4 and 5). This treatment was followed by onion oil and garlic oil, with respective disease incidence reductions of 79.4 and 81.1% in onion and 63.2 and 61.1% in garlic. The lowest reduction in white rot disease incidence was obtained with onion and garlic powders.

### 3.3 Effects on plant growth and bulb yield in the field

The sclerotial germination stimulants used in the absence of *Allium* crops affected the plant growth of onion cv. Giza 20 and garlic cv. Sides 40 in two field trials (Tables 6 and 7). The results indicate that the extent of growth improvement was related to the inoculum density. The mean growth parameters of plants in soil containing 45.9 sclerotia/kg were significantly greater than in soil containing 594.7 sclerotia/kg of soil. In general, germination stimulants were more effective in improving onion and garlic growth in the trial with the low inoculum density compared with the trial with the high inoculum density. In the low inoculum density trial for onion, all the tested germination stimulants were effective, increasing the average plant height by 22.9%–25.7%, increasing the average number of leaves/plant by 22.9–30.9% and increasing the average plant biomass by 27.7–33.2% (Table 6). The effects on garlic grown in soil with low inoculum density followed the same trend (Table 7). The effects of soil application with germination stimulants used in the absence of *Allium* crops on subsequent onion and garlic bulb yield at the two sites followed the same trend but the bulb yield was greater in soil with low inoculum density than that with high inoculum density (Table 8). The data in Table 8 reveal that all the tested germination stimulants used have pronounced effects in comparison with the control. In the low inoculum density trial, all the tested germination stimulants were effective, increasing the average bulb yield per plot by 31.9–37.7% in onion and by 29.8–34.2% in garlic (Table 8).

**Table 6:** Effects on average plant height, average number of leaves/plant and average plant biomass of onion plants due to application of sclerotial germination stimulants in the absent of *Allium* crops during the previous season under field conditions(a) at 100 days after planting.

Treatment	Onion plants grown in field		
	Plant height (cm)	Number of leaves/plant	Plant biomass (g)
**Trial I (45.9 sclerotia/kg soil)**			
Control	53.7 ± 0.9 b(b)	6.7 ± 0.3 b	74.0 ± 1.2 b
Onion powder	70.7 ± 0.7 a	9.3 ± 0.3 a	107.0 ± 5.7 a
Garlic powder	70.0 ± 1.2 a	8.7 ± 0.3 a	110.0 ± 1.2 a
Onion oil	71.7 ± 2.0 a	8.7 ± 0.3 a	102.3 ± 0.3 a
Garlic oil	69.7 ± 0.9 a	9.3 ± 0.3 a	103.0 ± 4.6 a
*Allium* waste	72.3 ± 0.3 a	9.7 ± 0.3 a	110.7 ± 2.4 a
**Trial II (594.7 sclerotia/kg soil)**			
Control	45.7 ± 0.9 a	5.7 ± 0.3 b	44.7 ± 1.8 c
Onion powder	48.0 ± 0.6 a	6.3 ± 0.3 ab	48.7 ± 0.3 ab
Garlic powder	46.7 ± 3.2 a	6.7 ± 0.3 ab	46.0 ± 0.6 bc
Onion oil	49.3 ± 0.9 a	6.3 ± 0.3 ab	48.7 ± 0.3 ab
Garlic oil	47.7 ± 0.9 a	6.7 ± 0.3 ab	50.0 ± 1.5 a
*Allium* waste	50.3 ± 0.4 a	7.0 ± 0.0 a	49.7 ± 0.7 a

(a) Sclerotial germination stimulants were added to soil in 1-12-2016. The garlic was sown in September 15, 2017 and onion was sown in December 1, 2017.

(b) Means ± standard errors within a column followed by the same letter are not significantly different by Duncan multiple range test at *P* < 0.05.

**Table 7:** Effects on average plant height, average number of leaves/plant and average plant biomass of garlic plants due to application of sclerotial germination stimulants in the absent of *Allium* crops during the previous season under field conditions(a) at 100 days after planting.

Treatment	Garlic plants grown in field		
	Plant height (cm)	Number of leaves/plant	Plant biomass (g)
**Trial I (45.9 sclerotia/kg soil)**			
Control	60.0 ± 1.2 b(b)	7.3 ± 0.3 b	68.7 ± 1.8 b
Onion powder	75.3 ± 0.3 a	9.0 ± 0.0 a	108.7 ± 1.8 a
Garlic powder	74.7 ± 0.9 a	9.3 ± 0.3 a	109.0 ± 2.1 a
Onion oil	77.7 ± 0.9 a	9.7 ± 0.3 a	100.3 ± 2.0 b
Garlic oil	74.7 ± 1.8 a	9.3 ± 0.3 a	101.7 ± 3.2 ab
*Allium* waste	78.3 ± 1.2 a	10.0 ± 0.0 a	106.7 ± 1.3 ab
**Trial II (594.7 sclerotia/kg soil)**			
Control	50.0 ± 0.6 c	6.3 ± 0.3 a	48.3 ± 2.0 b
Onion powder	50.7 ± 0.3 abc	6.7 ± 0.3 a	53.7 ± 1.5 a
Garlic powder	50.7 ± 0.3 abc	6.7 ± 0.3 a	53.7 ± 0.3 a
Onion oil	50.3 ± 0.3 bc	6.7 ± 0.3 a	53.7 ± 0.3 a
Garlic oil	52.0 ± 1.2 a	6.7 ± 0.3 a	54.3 ± 1.4 a
*Allium* waste	51.7 ± 0.9 ab	7.0 ± 0.0 a	54.7 ± 0.7 a

(a) Sclerotial germination stimulants were added to soil in 1-12-2016. The garlic was sown in September 15, 2017 and onion was sown in December 1, 2017.

(b) Means ± standard errors within a column followed by the same letter are not significantly different by Duncan multiple range test at *P* < 0.05.

**Table 8:** Effects on onion and garlic bulb yield (2017/2018 season) due to application of sclerotial germination stimulants in the absent of *Allium* crops during the previous season (2016/2017 season) under field conditions.(a)

Treatment	Onion and garlic bulb yield (kg/plot)			
	Trial I (45.9 sclerotia/kg soil)		Trial II (594.7 sclerotia/kg soil)	
	Onion	Garlic	Onion	Garlic
Control	14.3 ± 0.2 d(b)	16.0 ± 0.1 d	8.1 ± 0.1 b	8.1 ± 0.1 b
Onion powder	22.4 ± 0.1 a	23.1 ± 0.1 bc	8.8 ± 0.4 a	9.4 ± 0.2 a
Garlic powder	21.0 ± 0.0 c	23.5 ± 0.0 b	9.3 ± 0.1 a	9.7 ± 0.1 a
Onion oil	21.9 ± 0.4 ab	23.2 ± 0.2 bc	9.4 ± 0.1 a	9.6 ± 0.1 a
Garlic oil	21.6 ± 0.0 bc	22.8 ± 0.1 c	9.5 ± 0.1 a	9.1 ± 0.1 a
*Allium* waste	22.6 ± 0.3 a	24.3 ± 3.5 a	9.1 ± 0.1 a	9.3 ± 0.3 a

(a) Sclerotial germination stimulants were added to soil in 1-12-2016. The garlic was sown in September 15, 2017 and onion was sown in December 1, 2017.

(b) Means ± standard errors within a column followed by the same letter are not significantly different by Duncan multiple range test at *P* < 0.05.

### 3.4 Benefit-cost balance of sclerotial germination stimulants under field conditions

For onion and garlic crops, under low sclerotial density (45.9 sclerotia/kg of soil), field application with all sclerotial germination stimulants increased the income as compared with untreated controls (Tables 9 and 10). The highest income was obtained with *Allium* waste followed by onion powder. Application of garlic powder showed moderate effect. Moreover, under high sclerotial density (594.7 sclerotia/kg of soil), only *Allium* waste had little positive effect, while other stimulants had negative effect (Tables 9 and 10).

**Table 9:** The costs and benefits of onion bulb yield due to application of sclerotial germination stimulants in the absent of *Allium* crops during the previous season under field conditions.

Treatment	Onion					
	Costs(a) (L.E)	Yield without treatments (Ton/hectare)	Yield with treatments (Ton/hectare)	Saving (Ton)	Benefits(b) (L.E)	Saving (L.E)
**Trial I (45.9 sclerotia/kg soil)**						
Onion powder	5000	14.0	22.0	8.0	24000	19000
Garlic powder	5000	14.0	21.0	7.0	21000	16000
Onion oil	10000	14.0	21.9	7.0	21000	11000
Garlic oil	10000	14.0	21.0	7.0	21000	11000
*Allium* waste	900	14.0	22.0	8.0	24000	23100
**Trial II (594.7 sclerotia/kg soil)**						
Onion powder	5000	8.1	8.8	0.7	2100	−2900
Garlic powder	5000	8.1	9.3	1.2	3600	−1400
Onion oil	10000	8.1	9.4	1.3	3900	−6100
Garlic oil	10000	8.1	9.5	1.4	4200	−5800
*Allium* waste	900	8.1	9.1	1.0	3000	2100

(a) Costs included the price of the treatments and wages of labors.

(b) Benefits was calculated as average price/ton = 3000 LE.

**Table 10:** The costs and benefits of garlic bulb yield due to application of sclerotial germination stimulants in the absent of *Allium* crops during the previous season under field conditions.

Treatment	Garlic					
	Costs(a) (L.E)	Yield without treatments (Ton/hectare)	Yield with treatments (Ton/hectare)	Saving (Ton)	Benefits(b) (L.E)	Saving (L.E)
**Trial I (45.9 sclerotia/kg soil)**						
Onion powder	5000	16.0	23.1	7.1	21300	16300
Garlic powder	5000	16.0	23.5	7.5	22500	17500
Onion oil	10000	16.0	23.2	7.2	21600	11600
Garlic oil	10000	16.0	23.8	7.8	23400	13400
*Allium* waste	900	16.0	24.3	8.3	24900	24000
**Trial II (594.7 sclerotia/kg soil)**						
Onion powder	5000	8.1	9.4	1.3	3900	−1100
Garlic powder	5000	8.1	9.7	1.6	4800	−200
Onion oil	10000	8.1	9.6	1.5	4500	−5500
Garlic oil	10000	8.1	9.1	1.0	3000	−7000
*Allium* waste	900	8.1	9.3	1.2	3600	2700

(a) Costs included the price of the treatments and wages of labors.

(b) Benefits was calculated as average price/ton = 3000 LE.

## 4 Discussion

**Fig. 2:**
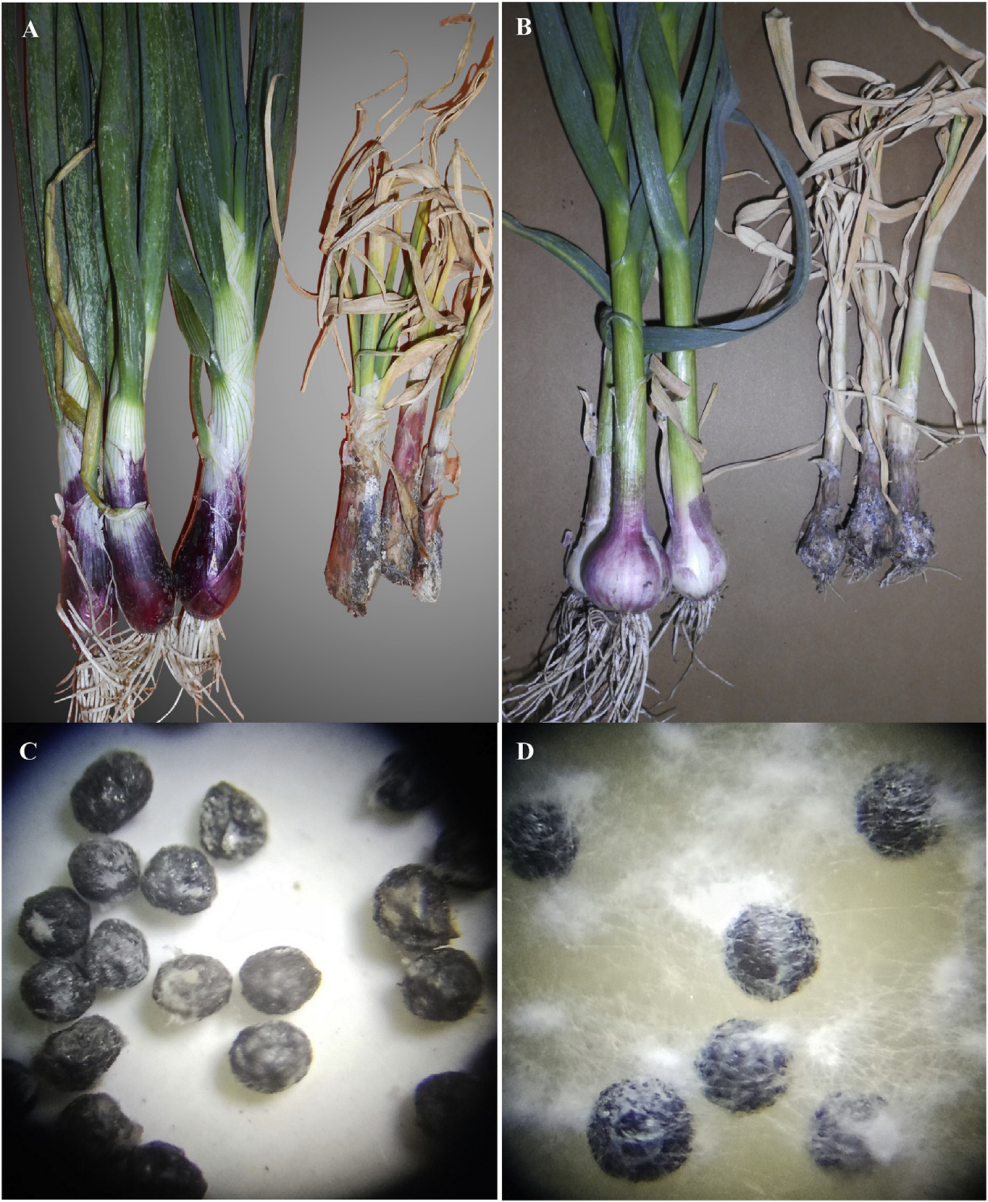
Onion (A) and garlic (B) plants showing healthy plants (left) and symptoms of white rot on infected plants (right). Sclerotia of *S. cepivora* formed from mycelium grown on infected plants (C) and that formed from mycelium culture grown on PDA medium (photos were taken with a dissecting microscope).

The first symptoms of *Allium*-white rot are yellowing and dying of the outer leaves of the plant, beginning at the tips and progressing downwards (Fig. 2). Roots and bases of scales are attacked and the fungus becomes evident on the infected bulbs as superficial white fluffy mycelium. The mycelium later produces sclerotia at the base of the plant (Fig. 2). During the harvest, sclerotia that formed within and on host tissue were distributed across the fields and through the soil profile. After its formation, the newly formed sclerotia remains dormant for several weeks or months by means of an unknown mechanism (Coley-Smith, 1960). After this period, these sclerotia germinate in the presence of volatile sulphides and thiols from the roots of *Allium* plants only (Coley-Smith, 1960, 1986; Coley-Smith and Parfitt, 1986). In the present research (laboratory and greenhouse experiments) demonstrated that soil treatments with sclerotial germination stimulants (*i.e*., onion powder, garlic powder, onion oil, garlic oil and *Allium* waste) reducing populations of viable sclerotia within 120 and 180 days after treatment. *Allium* waste, onion oil and garlic oil were the most effective, which reduced sclerotial populations by more than 90%. The powder of onion and garlic was apparently less effective than the other treatments. In the two field sites, one containing 45.9 sclerotia/kg and the other containing 594.7 sclerotia/kg, three applications of sclerotial germination stimulants also reduced the population of viable sclerotia 6 months after treatment. In general, the inoculum density of viable sclerotia was greatly reduced in all field plots in which germination stimulants were used, although the final population of viable sclerotia was much higher in soil containing 594.7 sclerotia/kg (202–313 sclerotia/kg) than in soil containing 45.9 sclerotia/kg (12.7–22.3 sclerotia/kg). The results obtained from the present study are consistent with previous reports of the effects of germination stimulants on the survival of sclerotia in treated mineral soils (Coley-Smith and Parfitt, 1986; Crow et al., 1994) and in muck soil (Terry, 1996). In the present study, sclerotia behaved similarly in all field trials. In both trials, two and four months after application, the population of viable sclerotia declined progressively. However, 6 months after application, a slight reduction was recorded may be due to soil temperature patterns. In the current study, the application of sclerotial germination stimulants during the winter months may have contributed to the rapid germination of sclerotia in the treated field. On 30/5/2017, soil temperature begins to rise under Egyptian conditions. Sclerotial germination in soil is stimulated between approximately 10–22 °C and may be restricted to the spring in some regions (Crowe and Hall, 1980; Gerbrandy, 1992). Crow et al. (1994) found that the sclerotial germination response is only highly efficient near the temperature and soil moisture optima for the fungus.

The efficacy of the sclerotial germination stimulants used in the absence of *Allium* crops on the incidence of white rot and on the yield of subsequent onion and garlic under field conditions was studied. Acceptable levels of onion white rot control were obtained by treating naturally infested field sites with germination stimulants for one consecutive year prior to growing onion and garlic. In the field site containing 45.9 sclerotia/kg, three applications of *Allium* waste, onion oil and garlic oil reduced white rot disease incidence to below 8.7% in onion and 17.3% in garlic at harvest. These results are consistent with previous reports in which germination stimulants in soil treatments reduced the incidence of *Allium* white rot on garlic and onion grown in mineral soils (Crowe et al., 1994; Juarez, 1998). However, despite the effectiveness of all stimulant treatments to reduce sclerotial populations in field sites containing 594.7 sclerotia/kg, the extent of disease in subsequent onion and garlic crops was very high. This may be due to high sclerotial density at planting. The average densities of viable sclerotia of *S. cepivora* in the soil at the date of planting, were 202–313 viable sclerotia/kg soil in the field containing 594.7 sclerotia/kg. In agreement with previous indications (Crowe et al., 1980; Entwistle, 1990), these results suggest a direct relationship between inoculum density and final disease incidence, where a high inoculum density corresponded to a high percentage of dead plants. Three different types of relationships between sclerotial populations of *S. cepivora* in the soil and the incidence of white rot have been reported (Entwistle, 1990), *i.e*., (1) sclerotia populations in soil and those needed for infection are both small, (2) soil populations of *S. cepivora* are large but small densities are required for infection, and (3) large populations are present and are needed for infection. Our results seem to fall within the third type, represented by white rot of onion and garlic in Egypt.

Plant health and bulb yield were positively affected by germination stimulants. These treatments significantly improved plant health *i.e.,* plant height, number of leaves/plant and plant biomass only in field trials containing 45.9 sclerotia/kg. Germination stimulants significantly increased onion and garlic yields compared to the non-treated control in the two field trials. Such results are in agreement with the earlier findings of Davis et al. (2007).

## 5 Conclusions

The ability of a soil-application of five sclerotial germination stimulants, *i.e.*, onion powder, garlic powder, onion oil, garlic oil and *Allium* waste to stimulate germination of sclerotia of *S. cepivora*, the cause of white rot disease of onion and garlic, was evaluated. In the field trials, survival of sclerotia decreased after 6 months of exposure to sclerotial germination stimulant-treated soil. Repeated applications with sclerotial germination stimulants reduced disease incidence in onion and garlic at harvest compared with the untreated controls in trials with low inoculum density (45.9 sclerotia/kg of soil). Despite the efficacy of the stimulants to reduce populations of sclerotia in soil with high inoculum density (594.7 sclerotia/kg of soil), the pathogen caused substantial white rot and yield losses in subsequent onion and garlic crops planted about approximately one year after soil treatment. Under low sclerotial density, field application with all sclerotial germination stimulants increased the income as compared with untreated controls. Moreover, under high sclerotial density, only *Allium* wastes had little positive effect, while other stimulants had negative effect.

## Declarations

### Author contribution statement

Ibrahim Elshahaway, Ahmed Morsy, Farid Abd-El-Kareem, Nehal Saied: Conceived and designed the experiments; Performed the experiments; Analyzed and interpreted the data; Contributed reagents, materials, analysis tools or data; Wrote the paper.

### Funding statement

This research did not receive any specific grant from funding agencies in the public, commercial, or not-for-profit sectors.

### Competing interest statement

The authors declare no conflict of interest.

### Additional information

No additional information is available for this paper.
